# Repeated Game Analysis of a CSMA/CA Network under a Backoff Attack

**DOI:** 10.3390/s19245393

**Published:** 2019-12-06

**Authors:** Juan Parras, Santiago Zazo

**Affiliations:** Information Processing and Telecommunications Center, Universidad Politécnica de Madrid, ETSI Telecomunicación, Av. Complutense 30, 28040 Madrid, Spain; santiago.zazo@upm.es

**Keywords:** CSMA/CA, backoff attack, repeated game, subgame perfect equilibrium, correlated equilibrium, Folk theorem

## Abstract

We study a CSMA/CA (Carrier Sense Medium Access with Collision Avoidance) wireless network where some stations deviate from the defined contention mechanism. By using Bianchi’s model, we study how this deviation impacts the network throughput and show that the fairness of the network is seriously affected, as the stations that deviate achieve a larger share of the resources than the rest of stations. Previously, we modeled this situation using a static game and now, we use repeated games, which, by means of the Folk theorem, allow all players to have better outcomes. We provide analytical solutions to this game for the two player case using subgame perfect and correlated equilibria concepts. We also propose a distributed algorithm based on communicating candidate equilibrium points for learning the equilibria of this game for an arbitrary number of players. We validate approach using numerical simulations, which allows comparing the solutions we propose and discussing the advantages of using each of the methods we propose.

## 1. Introduction

IEEE 802.11 [1] is a widespread standard used in wireless local area network communications. Each of the devices connected using this standard is known as station. Since the communication medium is shared among the stations, the Medium Access Control (MAC) layer regulates the medium access. In 802.11 standard, the medium access can be centralized, using Point Coordination Function (PCF, now obsolete) or Hybrid Coordinator Function (HCF); or distributed, using the Distributed Coordination Function (DCF), which uses CSMA/CA (carrier-sense medium access with collision avoidance). This mechanism is based on using a random backoff procedure designed to minimize the probability of collision among stations and is a popular choice not only in 802.11 standard but also in many other MAC layer protocols, such as Sensor MAC (SMAC) [2], WiseMAC [3], Timeout MAC (TMAC) [4] and Dynamic Sensor MAC (DSMAC) [5]. Indeed, CSMA is a popular choice when designing MAC protocols [6,7]. However, the deferral mechanism of CSMA/CA is vulnerable to backoff attacks, as a station may not follow the backoff procedure in order to obtain an advantage in terms of bandwidth [8].

An option to study these attacks consists in using game theory tools, which find many applications in the wireless networks field [9] and is a popular choice when it comes to multiple access attacks. In Reference [10], there is a survey on game theory approaches to multiple access situations in wireless networks and Reference [11] contains another survey more concretely focused on CSMA. Other works studying wireless networks under backoff attacks are References [12,13,14]. In Reference [13], the authors characterize two different families of Nash equilibria that arise in this situation and Reference [14] is devoted to the detection of such attacks. In Reference [12], the author proposes a strategy to face a selfish backoff behavior, called CRISP and provides simulations of its performance, although it assumes that all agents have the same target, assumption that we drop.

In this article, we continue the work presented in Reference [8], where we studied a CSMA/CA network under a backoff attack. The network throughput was estimated using Bianchi’s model [15], showing that a backoff attack did affect strongly to the network fairness, as misbehaving stations would achieve a larger portion of the network bandwidth at the expense of the stations that respected the backoff mechanism. We used a novel heterogeneous network model, in which we differentiate between attacking stations (ASs), whose objective is obtaining as much throughput as possible and a defense mechanism trying to fairly split the network throughput among all stations. We posed and solved that situation using game theory tools and taking into account the theoretical throughput decrease when the network was under attack, providing analytical solutions and a learning algorithm. However, we considered the game to be static, which means that we did not consider the influence of the time, as each station transmits more than once in real wireless network.

Thus, in this article, we take into account the effect of time in order to model the CSMA/CA backoff attack as a repeated game, a particularization of the more general class of stochastic games [16]. A repeated game is formed by repeatedly playing a static game [17] and a very interesting feature of repeated games is that the region of equilibria can be larger than the region of static equilibria. This phenomenon is collected by the Folk theorems [17,18]. These theorems provide conditions that, if satisfied, allow all players to obtain higher payoffs by using repeated game strategies instead of static game ones. In our game, this means that taking into account that there are several interactions among the stations may provide better payoffs for all players.

This work includes several features from Reference [8] which were not collected in previous approaches—we use a heterogeneous network model, which takes into account different aims between different types of stations in the network. We again use Bianchi’s model to estimate the effects of the bakoff on the network throughput. But now, we move to repeated game solutions, whereas Reference [8] only studied static ones. Thus, we provide the next important contributions:

- By using repeated games, and taking into account the time, we are able to use a more complex strategy which allows all players to obtain better payoffs, thanks to the Folk Theorem. Ours is not the first work that studies the CSMA/CA backoff attack using repeated games [12]. But to the best of our knowledge, is the first one that studies the backoff attack as a repeated game using average discounted payoff. As Reference [12] indicates, not taking into account a discount factor is not very realistic in a volatile environment as wireless networks and this approach have been used in other applications, such as smart grids [19].
- We do not focus on a single equilibrium concept but study the attack using both subgame perfect equilibria and correlated equilibria concepts and solve the game analytically for the two player case. This allows us comparing both equilibria concepts in terms of payoffs and computational capabilities required in the network stations and hence, we also include several guidelines in order to implement the approach described in this work.
- We also use a negotiation algorithm that allows finding solutions to the repeated game for more than two players and discuss its scalability and application in practical problems.

The rest of the article goes as follows. In Section 2, the DCF is described and Bianchi’s model is used to study the throughput under a backoff attack. Then, Section 3 gives a brief introduction to static and repeated games. Section 4 introduces the CSMA/CA problem and models it using game theory tools and Section 5 presents the solution to the static CSMA/CA game. Then, Section 6 solves the CSMA/CA game analytically using repeated game theory tools for the two players case and Section 7 provides an algorithm to solve it distributedly for an arbitrary number of players. Later, Section 8 presents several simulations where the payoff gain of using repeated game tools are shown, as well as the computational cost. This Section finishes comparing the different solutions proposed, providing some implementation guidelines. Finanly, in Section 9 we provide some conclusions. Section 2, Section 4, Section 5 and the first part of Section 3 are an overview of Reference [8] but they are included for the sake of clarity and completeness of the rest of the article.

## 2. Distributed Coordination Function in IEEE 802.11

### 2.1. Description of Basic Access Mechanism

As we have indicated, many MAC layer protocols are based on CSMA/CA. We now describe the well-known CSMA/CA mechanism implementation described in the DCF of the MAC layer in the 802.11 standard. The whole process can be a two-way handshaking in the Basic Access mechanism (BA) or a four-way handshaking in the Request-To-Send/Clear-To-Send mechanism (RTS/CTS). We focus only on BA, as it is widely used.

In BA mechanism, a station willing to transmit monitors the channel to determine whether it is idle, that is, no station transmits, or busy. If it is busy, the station defers the transmission until the channel is idle for a fixed period and then, starts a counter, called backoff, which decrements while the channel is idle. When the backoff counter reaches 0, the station transmits. This procedure minimizes the collision probability when the channel starts being idle after being busy, as different stations might be waiting to transmit.

The backoff counter follows a uniform random variable in the interval [0,CW−1], where CW stands for contention window. If a collision is detected when a station transmits, the value of CW is duplicated and the backoff procedure is repeated. The value of CW lies in the interval $\left[W,CW_{max}\right]$, where $CW_{max}=2^{m}W$, *m* is the maximum backoff stage and *W* is the minimum size of the contention window. This procedure is known as binary exponential backoff and it is the one used in IEEE 802.11 standard. Finally, after a successful transmission, the transmitting station waits for an acknowledgement frame: if none arrives, the station retransmits.

### 2.2. Network Throughput under Backoff Modification

A popular model to estimate the theoretical throughput in a CSMA/CA network is Bianchi’s model [15]. It assumes saturation of the network (i.e., that each station always has a packet to transmit) and that the probability of collision for a station is a constant. In References [8,12,13], it is shown that there might be ASs which modify their backoff to their advantage. Their impact on the network throughput can be analyzed using Bianchi’s model.

Assume that we have a network with *n* stations: $n_{1}$ normal stations (NSs) which follow the binary exponential backoff and $n_{2}=n-n_{1}$ ASs. For analytical tractability, we assume that ASs use a uniform backoff, in which their backoff counter follows a uniform random variable in the interval $\left[0,W_{2}-1\right]$. We can compute the collision probability $p_{i}$ for station *i* (i.e., the probability that station *i* observes a collision while transmitting a packet) and the probability that station *i* transmits a packet $\tau_{i}$ as the solution to [8,15]:

(1) $$\left\{\begin{array}{c} \tau_{1}=\frac{2}{1+W_{1}+p_{1}W_{1}\sum_{j=0}^{m_{1}-1}{\left(2p_{1}\right)}^{j}}\\ \tau_{2}=\frac{2}{1+W_{2}}\\ p_{1}=1-{\left(1-\tau_{1}\right)}^{n_{1}-1}{\left(1-\tau_{2}\right)}^{n_{2}}\\ p_{2}=1-{\left(1-\tau_{1}\right)}^{n_{1}}{\left(1-\tau_{2}\right)}^{n_{2}-1}, \end{array}\right.$$

where the subscript *i* denotes the class of a station and their parameters: $W_{1}$ and $m_{1}$ are the binary exponential backoff parameters of normal stations and $W_{2}$ the uniform backoff parameter for ASs. The solutions to (1) can be used to obtain the $S_{i}$, the throughput for station *i*, defined as the fraction of time used by station *i* to successfully transmit payload bits:

(2) $$S_{i}=\frac{T_{p}}{T_{slot}}\tau_{i}{\left(1-\tau_{i}\right)}^{n_{i}-1}\left(1-\tau_{j}\right),$$

where i∈{1,2} denotes the class of station, whether attacking or normal and j∈{1,2} denotes the opposite station class with respect to *i*. $T_{slot}$ is the expected duration of a slot time, which is related to the time used to count down a backoff unit, which is defined by IEEE 802.11 standard and denoted by $T_{s}$; to the time duration of a successful transmission ($T_{t}$); to the time duration of a collision ($T_{c}$) and to the time duration of the payload bits ($T_{p}$). Assuming that all these time intervals are the same for all stations, we have that [8]:

(3) $$\begin{array}{cc} T_{slot} & =\left(1-P_{tr}\right)T_{s}+\left(n_{1}P_{s,1}+n_{2}P_{s,2}\right)T_{t}+P_{c}T_{c}\\ T_{c} & =H+T_{p}+DIFS+T_{\delta }\\ T_{t} & =H+T_{p}+SIFS+T_{\delta }+ACK+DIFS+T_{\delta }, \end{array}$$

where *H* is the total header transmission time (adding PHY and MAC layers headers), DIFS and SIFS are interframe spacing, ACK is the transmission time of an ACK and $T_{\delta }$ is the propagation delay. All these parameters are defined in the 802.11 standard.

The rest of parameters in (2) and (3) are obtained using (1): $P_{tr}$ is the probability that there is at least one station transmitting, $P_{s,i}$ is the probability that there is exactly one station of class *i* transmitting and $P_{c}$ is the collision probability (i.e., the probability of two or more stations transmitting at once). These probabilities are [8]:

(4) $$\begin{array}{cc} P_{tr} & =1-\prod_{i=1}^{n}\left(1-\tau_{i}\right)=1-{\left(1-\tau_{1}\right)}^{n_{1}}{\left(1-\tau_{2}\right)}^{n_{2}}\\ P_{s,1} & =\tau_{1}{\left(1-\tau_{1}\right)}^{n_{1}-1}{\left(1-\tau_{2}\right)}^{n_{2}}\\ P_{s,2} & =\tau_{2}{\left(1-\tau_{1}\right)}^{n_{1}}{\left(1-\tau_{2}\right)}^{n_{2}-1}\\ P_{c} & =P_{tr}-n_{1}P_{s,1}-n_{2}P_{s,2}. \end{array}$$

Finally, the total network throughput, defined as the fraction of the time spent by all the stations transmitting successfully payload bits, is obtained using (2) as:

(5) $$S=\sum_{i}S_{i}=n_{1}S_{1}+n_{2}S_{2}.$$

Equations (1) to (5) are used in Reference [8] to study the impact of having $n_{2}$ stations that follow a uniform backoff. A main conclusion is that the throughput of the normal stations decreases significantly when there are ASs with a low value of $W_{2}$. Intuitively, this happens because the ASs use lower backoffs and hence, they have higher chances to win the contention procedure against normal stations. Actually, it is shown than a single AS may use more than half of the total transmission time of the network. Thus, it is important to study this situation, in order to avoid a small set of ASs excessively using the network resources illegitimately: we use game theory tools for this purpose.

## 3. Introduction to Game Theory

We provide a brief introduction to static and repeated games. More exhaustive treatments are given in References [17,18,20,21].

### 3.1. Static Games

We define a static game as follows [20]:

**Definition** **1** (Static game)**.** *A static game G is a triple $\langle N_{p},A,u\rangle$, where:*

- $N_{p}$ is the number of players, numbered as $1,\ldots ,N_{p}$.
- A is the set of actions available to all players. The pure actions available to player i are denoted by $a_{i}$, with $a_{i}\in A_{i}$, being $A_{i}$ the set of actions available to player i. A is defined as $A\equiv \prod_{i}A_{i}$. A is assumed to be a compact (i.e., bounded and closed) subset of $R^{N_{p}}$.
- u is a continuous function that gives the game payoffs:

(6) $$u:\prod_{i}A_{i}\to R^{N_{p}}.$$

We use discrete sets of actions (i.e., $A_{i}$ are finite sets) and each of these actions are pure actions. If there are $N_{p}=2$ players, the payoff functions for each player can be expressed using a matrix $R_{i}$, whose dimensions are the number of actions of each player: entry $r_{ij}$ is the payoff when row player chooses its action *i* and column player its action *j*. If the sum of the payoff of the players equals zero, that is, $\sum_{i}u_{i}\left(a\right)=0,\forall a\in A$, the game is known as zero-sum game: note that the gains of some players are the loses of the others and hence, zero-sum games model situations of extreme competition among players. If the sum of the payoffs is different from zero, the game is called non-zero sum game.

### 3.2. Repeated Games of Perfect Monitoring

A repeated game is built using a static game, which is played repeatedly over several periods. This static game is called stage game. We work with repeated games of infinite horizon: the stage game is played on the periods t∈{0,1,2,…,+∞}. The main elements in a repeated game are the followings, where superscript indicates time and subscript indicates the players [18]:

1. The set of histories $H^{t}$. A history $h^{t}$ is a list of the action profiles played in periods {0,1,…,t−1}. Thus, the history contains the past actions.
2. A strategy for player *i* is a mapping from the set of all possible histories into the set of actions: $\sigma_{i}:H\to A_{i}$.
3. Continuation: for any history $h^{t}$, the continuation game is the infinitely repeated game that begins in period *t*, following history $h^{t}$. After playing up to time *t*, a strategy must consider all possible continuation histories $h^{\tau }$ and be a strategy for each possible $h^{\tau }$ or equivalently, for each concatenation of histories $h^{t}h^{\tau }$. In other words, a strategy must depend only on the previous history.
4. The average discounted payoff to player *i* is given by:
  (7) $$V_{i}\left(\sigma \right)=\left(1-\delta \right)\sum_{t=0}^{\infty }\delta^{t}u_{i}\left(a^{t}\left(\sigma \right)\right),$$
  where δ is the discount factor, satisfying δ∈[0,1). Note that the payoff $V_{i}$ is normalized with the term 1−δ, which allows comparing payoffs in the repeated game with the ones in the stage game.

We consider only repeated games of perfect monitoring, in which the history $h^{t}$ is known to all players, that is, all players observe the actions of the others at the end of each stage. In order to keep notation clear, we use $u_{i}$ to denote static equilibrium payoffs or stage games payoff and $V_{i}$ to denote the averaged discounted payoff of a repeated game.

## 4. Problem Description

**Figure 1:**
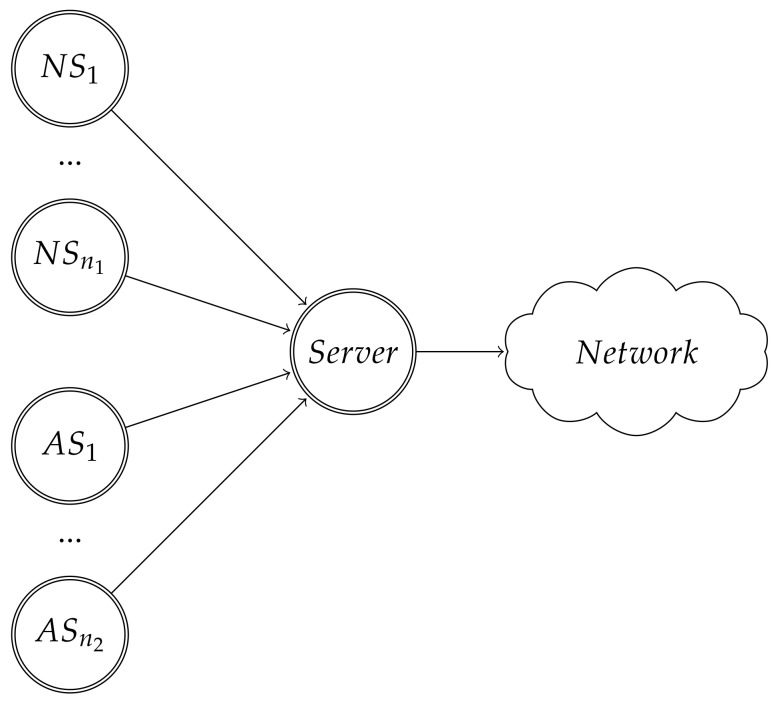
Network scheme for the case that there are $n_{1}$ normal stations (NS) and $n_{2}$ attacking stations (AS). NS respect 802.11 binary exponential backoff, whereas AS can choose to use it or to use a uniform backoff. Extracted from Reference [8].

We showed in Section 2.2 that, in a network using CSMA/CA, if some stations do not follow the established backoff procedure, the throughputs of the stations would not be evenly distributed. We study this problem using game theory tools, as done in Reference [8]. We use the network schema from Figure 1, with *n* stations: $n_{1}$ NSs which always follow the binary exponential backoff; and $n_{2}=n-n_{1}$ ASs which can choose between using the binary exponential backoff or the uniform backoff. All *n* stations are connected to a gateway, called server, which forwards their packets to a network. We only consider the uplink in the problem: the stations try to send packets to the server.

The players of the game are the server and the ASs; thus there are $N_{p}=n_{2}+1$ players. Each AS tries to maximize its throughput, whereas the server tries to enforce that all stations obtain a fair throughput (i.e., no station is getting a higher throughput at expense of others) by detecting misbehavior. If the server detects a station modifying its backoff, it drops the packet sent by that station: the station will have to retransmit and that decreases its throughput. We assume that the server is able to perfectly detect misbehavior of the stations, although that detection has a cost to the server, in terms of delay in the forwarding of the package and computational resources. Regarding the detection mechanism, there are many possible choices that could be used, such as the ones presented in References [12] or [14].

Each player has two actions: the ASs can behave selfishly (*s*) by using the uniform backoff or not (ns) by using the binary exponential backoff. As the procedure to test whether a station is an AS or an NS has a cost, the server can choose to perform the detection test (*d*) or not (nd).

We use Equations (1) to (5) to obtain the throughput values in our particular setup. We denote as $S_{j}^{a},j\in \left\{1,2,\ldots ,n_{2}\right\}$ the throughput that AS *j* obtains when action *a* is played and $S_{n_{1}}^{a}$ is the throughput that each NS obtains when action *a* is played; *a* is a vector of pure actions for all players. We follow Reference [8] and model the payoff functions as linear functions of the throughput. For the two player case (i.e., $n_{2}=1$), the payoff functions obtained are in Table 1 and can be simplified to the following payoff matrices:

(8) $$R_{1}=\left(\begin{array}{cc} -\alpha_{m} & 0\\ \alpha_{c} & -\alpha_{f} \end{array}\right)\;R_{2}=\left(\begin{array}{cc} \beta_{s} & 0\\ -\beta_{c} & 0 \end{array}\right),$$

where $\alpha_{c},\alpha_{m},\alpha_{f},\beta_{s},\beta_{c}\in \left(0,+\infty \right)$. It is possible to observe that the CSMA/CA game is a non-zero sum game. We also remark that, by using payoff matrices to solve the game in the incoming Sections, our model can be adapted to accommodate payoff matrices that are related to other network performance metrics, such as delay [8].

## 5. CSMA/CA Static Game

In this section, we introduce two equilibrium concepts for static games and apply them to the CSMA/CA game when $N_{p}=2$. We also include an algorithm that can be used to learn a static equilibrium for an arbitrary number of players.

### 5.1. Nash Equilibrium Concept

A well-known solution concept for games is the Nash equilibrium (NE). An NE is a vector of actions such that no player can obtain a better payoff by a unilateral deviation. Every non-zero sum game has at least one NE in mixed actions [20]. In a mixed equilibrium, each player has access to a randomizing device which outputs a certain pure action that the player should play, where the probability of each action is the mixed NE.

The CSMA/CA game posed in Section 4 can be solved using the NE concept. We define *y* as the probability that the server plays nd, thus 1−y is the probability that it plays *d*. For the AS, *z* is the probability of playing *s* and 1−z the probability of playing ns. The CSMA/CA has the following unique mixed NE [8]:

(9) $$y_{n}=\frac{\beta_{c}}{\beta_{c}+\beta_{s}},\;z_{n}=\frac{\alpha_{f}}{\alpha_{f}+\alpha_{m}+\alpha_{c}},$$

where $y_{n}$ and $z_{n}$ denote the mixed NE. The expected payoff that each player obtains if they play mixed actions with probability (y,1−y) for the server and (z,1−z) for the AS are:

(10) $$\begin{array}{cc} u_{1}\left(y,z\right)= & \left(y,1-y\right)R_{1}{\left(z,1-z\right)}^{T}=-zy\left(\alpha_{m}+\alpha_{c}+\alpha_{f}\right)+z\left(\alpha_{c}+\alpha_{f}\right)+\alpha_{f}\left(y-1\right)\\ u_{2}\left(y,z\right)= & \left(y,1-y\right)R_{2}{\left(z,1-z\right)}^{T}=zy\left(\beta_{s}+\beta_{c}\right)-z\beta_{c}, \end{array}$$

and thus, the expected NE payoffs using (9) are:

(11) $$u_{1}=-\frac{\alpha_{f}\alpha_{m}}{\alpha_{m}+\alpha_{c}+\alpha_{f}},\;u_{2}=0.$$

From (11), we observe that the payoff of the AS is 0 regardless of the parameters in (8). That means that the AS always obtains a payoff better or equal as if it behaved as a normal station. But the payoff of the server depends on the values in (8), moreover, $u_{1}$ will always be negative: the server always has a loss.

### 5.2. Correlated Equilibrium Concept

Another well-known equilibrium concept is the correlated equilibrium (CE), which generalizes NE [22]—every NE is a CE but not every CE is an NE. CE uses a correlating device, which produces a signal following a certain distribution ϕ(a) over the set of joint pure actions of the players $A=\;A_{1}\;\times \;A_{2}\;\times \;\ldots \;\times \;A_{N_{p}}$, where $a=(a_{1},a_{2},\ldots ,a_{N_{p}})$ is a vector of pure actions such that a∈A. This signal coordinates all players, as it says which pure action each player should use. A CE is a ϕ(a) vector such that no player can obtain a better payoff by deviating. Mathematically, the equilibrium condition for each player is [17,22]:

(12) $$\begin{array}{cc} \sum_{a_{-i}\in A_{-i}}\phi \left(a_{-i}|a_{i}\right)u_{i}\left(a_{i},a_{-i}\right) & \geq \sum_{a_{-i}\in A_{-i}}\phi \left(a_{-i}|a_{i}\right)u_{i}\left(a_{i}^{\prime },a_{-i}\right)\;\forall a_{i}^{\prime }\in A_{i},\;a_{i}\neq a_{i}^{\prime }, \end{array}$$

where $A_{-i}$ is the set of joint pure actions of all players except player *i*. An important advantage of CE is that they are less expensive to compute than NE [23,24].

The CSMA/CA game can be solved using the CE concept. Applying (12) as it is shown in Reference [8], there is only one CE in the CSMA/CA game, which coincides with the NE:

(13) $$\begin{array}{cc} \phi_{11} & =\frac{\alpha_{f}}{\alpha_{c}+\alpha_{m}+\alpha_{c}}\frac{\beta_{c}}{\beta_{c}+\beta_{s}}\\ \phi_{12} & =\frac{\alpha_{c}+\alpha_{m}}{\alpha_{c}+\alpha_{m}+\alpha_{c}}\frac{\beta_{c}}{\beta_{c}+\beta_{s}}\\ \phi_{21} & =\frac{\alpha_{f}}{\alpha_{c}+\alpha_{m}+\alpha_{c}}\frac{\beta_{s}}{\beta_{c}+\beta_{s}}\\ \phi_{22} & =\frac{\alpha_{c}+\alpha_{m}}{\alpha_{c}+\alpha_{m}+\alpha_{c}}\frac{\beta_{s}}{\beta_{c}+\beta_{s}}. \end{array}$$

The expected payoff provided by NE and CE is the same, following the expression in (11), as shown in Reference [8].

### 5.3. Learning Algorithms: Regret Matching

The static equilibria can also be learned. A simple and well-known algorithm used to learn static equilibria is Regret Matching (RM), proposed by Hart and Mas-Colell [25,26]. It assumes that each player only knows her payoff and can observe the actions of the rest of the players and also it assumes that the static game is played many times. Each player acts following a distribution which is updated each time that the static game is played. The update is done using a regret measure: the benefit that the player would have had in the past if she had played another action. Hence, it is an adaptive strategy which converges to the set of CE of the static game if all players use this kind of strategies [26].

It is important to note that even though RM learns in a repeated game, it learns a static equilibrium. We know that a static equilibrium is also a valid equilibrium in the repeated game but the Folk theorems assert that this static equilibrium needs not give the best payoffs achievable.

## 6. CSMA/CA Repeated Game in the Two Player Case

Now, we solve the CSMA/CA game treating it as a repeated game in the two player case.

### 6.1. Subgame Perfect Equilibrium Concept

An NE is the best response to the strategies of other players, as we saw in Section 5.1. NE concept can be extended to repeated games. The main difference with the static case is that the NE in a repeated game is defined in terms of the averaged discounted payoff (7) and the game solutions are optimal strategies. In repeated games, NE is strengthened by imposing the sequential rationality requirement: the behavior followed by the players must be optimal in all circumstances [18]. This gives rise to the Subgame Perfect Equilibrium (SPE)—a strategy profile σ is an SPE if it is an NE for every possible subgame of the repeated game.

Checking whether a concrete strategy profile σ is an SPE might become intractable, as there are infinity possible deviations. This is simplified by grouping the histories into equivalence classes: sets of histories that induce an identical continuation strategy. This allows describing the strategy using an automaton $\left(W,w^{0},f,\tau \right)$ [18], where:

- W is a set of states (each state is an equivalence class).
- $w^{0}\in W$ is the initial state.
- f:W→A is a decision function that maps states to actions, where $f\left(h^{t}\right)=\sigma \left(h^{t}\right)$.
- τ:W×A→W is a transition function that identifies the next state of the automaton as a function of the present state and the realized action profile, where $\tau \left(h^{t},a\right)=h^{t+1}$. A state is accessible from another state if the transition function links both states with some action.

The advantage of using an automaton is that often, the set of states W is finite, whereas the set of histories is not. Also, the automaton definition allows defining the averaged discounted payoff for player *i* in a game that starts in state *w* using Bellman’s equation as:

(14) $$V_{i}\left(w\right)=\left(1-\delta \right)u_{i}\left(a\right)+\delta V_{i}\left(\tau \left(w,a\right)\right).$$

In case of using mixed strategies, we take mathematical expectations in Equation (14). $V_{i}\left(w\right)$ is called continuation promise. A continuation promise is credible if, for each player and state, $V_{i}\left(w\right)\;\geq \;\left(1-\delta \right)u_{i}\left(a_{i}^{\prime },a_{-i}\right)+\delta V_{i}\left(\tau \left(w,\left(a_{i}^{\prime },a_{-i}\right)\right)\right),\forall a_{i}^{\prime }\neq a_{i}$. That is, is credible if it is an equilibrium. This allows treating repeated games as static games in order to solve them, as the next proposition taken from Reference [18] shows:

**Proposition** **1.** *Suppose that a strategy profile σ is described by an automaton $\left(W,w^{0},f,\tau \right)$. The strategy profile σ is an SPE if and only if for all w∈W accessible from $w^{0}$, f(w) is a Nash equilibrium of the normal form game described by the payoff functions $g^{w}:A\to R_{p}^{N}$ where*

$$g_{i}^{w}\left(a\right)=\left(1-\delta \right)u_{i}\left(a\right)+\delta V_{i}\left(\tau \left(w,a\right)\right).$$

In other words, we can test a strategy σ by obtaining the equivalent static game described with payoffs $g^{w}$ and checking for existence of NE. We use the following approach to obtain an SPE [18]: we fix a strategy in advance and then use Proposition 1 to check whether this strategy yields an equilibrium to the game. One possible candidate strategy would be always playing a static NE of the stage game. Proposition 1 shows that the players would obtain their static Nash payoff, independently of the value of δ. Hence, we have the same payoff that we had in the static case (Section 5): the stage NE is also an SPE in the repeated game.

However, this payoff could be improved, as the Folk theorems assert [17,18]. Roughly speaking, the Folk theorems state that in a repeated game, for a δ value sufficiently close to 1, any feasible payoff can be achieved, not only the static NE of the stage game. The discount factor gives a measure on how “patient" a player will be, meaning how much weight a player puts on future payoffs when compared to the actual payoff. Intuitively, the Folk theorems state that a player patient enough is able to obtain better payoffs. A repeated game may have infinitely many strategies that are an SPE and that yield payoffs equal or better than the static Nash payoff to every player.

There are many well-known strategies that are used to take advantage of the Folk theorems, such as Nash reversion, tit-for-tat, grim trigger or forgiving strategies [18,27]. All these strategies agree on a strategy that all players should follow and a punishment strategy which arises if any of the players deviate from the agreed strategy. Hence, the ability to obtain better payoffs by taking into account future play is closely related to being able to detect deviations instantaneously. This means that we have perfect monitoring: all players perfectly observe the actions of the other players. In case of mixed actions, this means the output of the randomizing device of the players is observed by other players.

In this article, we use as strategy unforgiving Nash reversion (UNR): both players start playing an agreed strategy $\left(y_{o},z_{o}\right)$ that provides them a payoff higher than their stage Nash payoff. If a deviation is observed, all players switch to play strategy $\left(y_{n},z_{n}\right)$, their stage NE strategy (obtained in Section 5.1). This punishment phase lasts forever, that is: if a player deviates, all players switch to play their stage NE strategy indefinitely. We choose UNR strategy because it is a simple strategy, with low computational requirements and hence, suitable for sensor networks. Nonetheless, as our simulations show, this strategy allows all players to improve their payoffs by making use of Folk Theorem tools.

### 6.2. SPE Solution to the CSMA/CA Game

Let us solve the CSMA/CA game using the ideas from Section 6.1. We start demonstrating the validity of UNR strategy with the server, using Proposition 1 and the expected payoff values from (10). UNR strategy is an SPE for the server if:

(15) $$\left(1-\delta \right)u_{1}\left(y_{o},z_{o}\right)+\delta V_{1}\left(y_{o},z_{o}\right)\geq \left(1-\delta \right)u_{1,max}\left(y,z_{o}\right)+\delta V_{1,n},$$

where $u_{1,max}\left(y,z_{o}\right)$ is the maximum payoff that the server can obtain from a unilateral deviation, $V_{1}\left(y_{o},z_{o}\right)$ is the payoff that the server expects to obtain by playing $y_{o}$ when the AS plays $z_{o}$ and $V_{1,n}$ is the payoff that the server expects to obtain if it deviates, which is the stage NE payoff. Observe that $V_{1}\left(y_{o},z_{o}\right)$ is the payoff if both players follow the UNR strategy without deviation, that is, $V_{1}\left(y_{o},z_{o}\right)\;=\;u_{1}\left(y_{o},z_{o}\right)$. Hence, (15) becomes:

(16) $$u_{1}\left(y_{o},z_{o}\right)\geq \left(1-\delta \right)u_{1,max}\left(y,z_{o}\right)+\delta V_{1,n},$$

which means that the discount factor must satisfy:

(17) $$\delta \geq \frac{u_{1,max}\left(y,z_{o}\right)-u_{1}\left(y_{o},z_{o}\right)}{u_{1,max}\left(y,z_{o}\right)-V_{1,n}},\;u_{1,max}\left(y,z_{o}\right)>V_{1,n}.$$

Now, we turn to the AS. We know that the stage NE payoff for the AS is $V_{2,n}=0$. Hence, UNR strategy is an SPE for the AS if:

(18) $$u_{2}\left(y_{o},z_{o}\right)\geq \left(1-\delta \right)u_{2,max}\left(y_{o},z\right),$$

which means that the discount factor must satisfy:

(19) $$\delta \geq 1-\frac{u_{2}\left(y_{o},z_{o}\right)}{u_{2,max}\left(y_{o},z\right)},\;u_{2,max}\left(y_{o},z\right)>0$$

Hence, from (17) and (19), UNR strategy is an SPE strategy for the CSMA/CA game if the following set of conditions are satisfied:

(20) $$\begin{array}{cc} \delta & \geq \max\left(\frac{u_{1,max}\left(y,z_{o}\right)-u_{1}\left(y_{o},z_{o}\right)}{u_{1,max}\left(y,z_{o}\right)-V_{1,n}},1-\frac{u_{2}\left(y_{o},z_{o}\right)}{u_{2,max}\left(y_{o},z\right)}\right)\\ \;\delta & \in \left[0,1\right),\;u_{1,max}\left(y,z_{o}\right)>V_{1,n},\;u_{2,max}\left(y_{o},z\right)>0. \end{array}$$

Observe that if players followed UNR without deviating, their payoff would be $\left(V_{1}\left(y_{o},z_{o}\right),V_{2}\left(y_{o},z_{o}\right)\right)=\left(u_{1}\left(y_{o},z_{o}\right),u_{2}\left(y_{o},z_{o}\right)\right)$. Both players must choose the strategy values $\left(y_{o},z_{o}\right)$ so that the conditions from (20) are satisfied. It might happen that $\left(y_{o},z_{o}\right)=\left(y_{n},z_{n}\right)$ (i.e., no UNR strategy gives higher payoff than stage NE) or that there is one or more valid $\left(y_{o},z_{o}\right)\neq \left(y_{n},z_{n}\right)$: this problem might have multiple solutions.

We consider that $u_{1,max}\left(y,z_{o}\right)$, the maximum payoff for the server if it deviates (equivalently, $u_{2,max}\left(y_{o},z\right)$ for the AS) is the expected payoff of deviating by using the mixed action *y* in case of the server (and *z* in case of the AS). After we have fixed $u_{1}\left(y_{o},z_{o}\right)$ and $u_{2}\left(y_{o},z_{o}\right)$, we compute $y_{o}$ and $z_{o}$ using (10) and then, we use (10) again in order to obtain $u_{1,max}\left(y,z_{o}\right)$ and $u_{2,max}\left(y_{o},z\right)$ as the solutions to:

(21) $$\begin{array}{cc} u_{1,max}\left(y,z_{o}\right) & =\max_{y}u_{1}\left(y,z\right)\;s.t.\;z=z_{o}\\ u_{2,max}\left(y_{o},z\right) & =\max_{z}u_{2}\left(y,z\right)\;s.t.\;y=y_{o}, \end{array}$$

whose solution, using (10), is:

(22) $$\begin{array}{cc} u_{1,max} & =\left\{\begin{array}{ccc} z_{o}\left(\alpha_{f}+\alpha_{c}\right)-\alpha_{f} & \mathrm{if} & z_{o}>z_{n}\\ -z_{o}\alpha_{m} & \mathrm{if} & z_{o}<z_{n} \end{array}\right.\\ u_{2,max} & =\left\{\begin{array}{ccc} y_{o}\left(\beta_{s}+\beta_{c}\right)-\beta_{c} & \mathrm{if} & y_{o}>y_{n}\\ 0 & \mathrm{if} & y_{o}<y_{n}. \end{array}\right. \end{array}$$

### 6.3. Correlated Equilibrium Concept

In the repeated game case, it is also possible to use the CE concept. We use the same idea that lies behind Proposition 1 as in Reference [28]. We define a static game which is equivalent to the repeated game using Bellman’s equation, as in (14). The automaton representation holds in the CE case with minor modifications, the main difference regarding the SPE case being that now we use the CE condition [18].

Again, we reduce the repeated game to a static one and solve it using the CE condition. We also use UNR strategy: both players commit to play a certain strategy ϕ until one deviates. If a deviation happens, the stage NE strategy is played. The set of CE is a convex set and there are algorithms that can approximate this set [29]. The strategies ϕ must satisfy (12), which for repeated games of perfect monitoring becomes:

(23) $$\begin{array}{cc} \sum_{a_{-i}\in A_{-i}}\phi \left(a_{-i}|a_{i}\right)V_{i}\left(a_{i},a_{-i}\right)\geq \sum_{a_{-i}\in A_{-i}} & \phi \left(a_{-i}|a_{i}\right)V_{i}\left(a_{i}^{\prime },a_{-i}\right)\;\forall a_{i}^{\prime }\in A_{i},\;a_{i}\neq a_{i}^{\prime }, \end{array}$$

where we use Bellman’s equation to define the payoff of the players as follows:

(24) $$V_{i}\left(a_{i},a_{-i}\right)=\left(1-\delta \right)u_{i}\left(a_{i},a_{-i}\right)+\delta V_{i}^{\prime }\left(a_{i},a_{-i}\right),$$

where $\left(a_{i},a_{-i}\right)$ is the vector containing the actions of the players, $V_{i}\left(a_{i},a_{-i}\right)$ is the expected payoff for player *i* if she plays $a_{i}$ and the rest of players play $a_{-i}$. This payoff has two components: the immediate payoff $u_{i}\left(a_{i},a_{-i}\right)$ and the future payoff $V_{i}^{\prime }\left(a_{i},a_{-i}\right)$. Observe that, for the sake of clarity, we drop the explicit use of τ and *w* regarding the notation form (14) but as we pointed out, the main change with respect to the NE case lies in using the CE condition, not in the notation.

### 6.4. Correlated Equilibrium Solution to the CSMA/CA Game

We compute the CE of the CSMA/CA game, using (23) and (24). We consider UNR strategy: both players will commit to use a strategy that yields a payoff $V_{o}=\left(V_{1,o},V_{2,o}\right)$ and if one of the players deviates, the other switches to its stage NE strategy, which yields a payoff $V_{n}=\left(V_{1,n},V_{2,n}\right)$. The CE condition, thus, using (23) becomes:

(25) $$\begin{array}{cc} \sum_{a_{2}=\left\{s,ns\right\}}\phi \left(a_{2}|d\right)V_{1}\left(d,a_{2}\right) & \geq \sum_{a_{2}=\left\{s,ns\right\}}\phi \left(a_{2}|d\right)V_{1}\left(nd,a_{2}\right)\\ \sum_{a_{2}=\left\{s,ns\right\}}\phi \left(a_{2}|nd\right)V_{1}\left(nd,a_{2}\right) & \geq \sum_{a_{2}=\left\{s,ns\right\}}\phi \left(a_{2}|nd\right)V_{1}\left(d,a_{2}\right)\\ \sum_{a_{1}=\left\{d,nd\right\}}\phi \left(a_{1}|s\right)V_{2}\left(s,a_{1}\right) & \geq \sum_{a_{1}=\left\{d,nd\right\}}\phi \left(a_{1}|s\right)V_{2}\left(ns,a_{1}\right)\\ \sum_{a_{1}=\left\{d,nd\right\}}\phi \left(a_{1}|ns\right)V_{2}\left(ns,a_{1}\right) & \geq \sum_{a_{1}=\left\{d,nd\right\}}\phi \left(a_{1}|ns\right)V_{2}\left(s,a_{1}\right). \end{array}$$

Using (8) and (24) and considering that $V_{i}^{\prime }=V_{i,o}$ if there is no deviation and $V_{i}^{\prime }=V_{i,n}$ if there is a deviation, the expressions in (25) become:

(26) $$\begin{array}{c} \left(\left(1-\delta \right)\alpha_{c}+\delta V_{1,o}\right)\phi \left(s|d\right)+\left(-\left(1-\delta \right)\alpha_{f}+\delta V_{1,o}\right)\phi \left(ns|d\right)\geq \\ \left(-\left(1-\delta \right)\alpha_{m}+\delta V_{1,n}\right)\phi \left(s|d\right)+\left(0+\delta V_{1,n}\right)\phi \left(ns|d\right)\\ \left(-\left(1-\delta \right)\alpha_{m}+\delta V_{1,o}\right)\phi \left(s|nd\right)+\left(0+\delta V_{1,o}\right)\phi \left(ns|nd\right)\geq \\ \left(\left(1-\delta \right)\alpha_{c}+\delta V_{1,n}\right)\phi \left(s|nd\right)+\left(-\left(1-\delta \right)\alpha_{f}+\delta V_{1,n}\right)\phi \left(ns|nd\right)\\ \left(-\left(1-\delta \right)\beta_{c}+\delta V_{2,o}\right)\phi \left(d|s\right)+\left(\left(1-\delta \right)\beta_{s}+\delta V_{2,o}\right)\phi \left(nd|s\right)\geq \\ \left(0+\delta V_{2,n}\right)\phi \left(d|s\right)+\left(0+\delta V_{2,n}\right)\phi \left(nd|s\right)\\ \left(0+\delta V_{2,o}\right)\phi \left(d|ns\right)+\left(0+\delta V_{2,o}\right)\phi \left(nd|ns\right)\geq \\ \left(-\left(1-\delta \right)\beta_{c}+\delta V_{2,n}\right)\phi \left(d|ns\right)+\left(\left(1-\delta \right)\beta_{s}+\delta V_{2,n}\right)\phi \left(nd|ns\right) \end{array}.$$

We know that the following is satisfied:

(27) $$\begin{array}{c} \phi \left(a|b\right)=\frac{\phi (a\cap b)}{\phi (b)},\;\phi \left(a\cap b\right)=\phi \left(b\cap a\right), \end{array}$$

thus, we use (27) to simplify (26). We will use the following notation: $\phi_{11}=\phi \left(nd\cap s\right)$, $\phi_{12}=\phi \left(nd\cap ns\right)$, $\phi_{21}=\phi \left(d\cap s\right)$ and $\phi_{22}=\phi \left(d\cap ns\right)$. This is the joint distribution probability, considering that the first subscript refers to the pure action of the server and the second, to the pure action of the AS. We consider that pure action 1 for the server is nd and pure action 2, *d*; for the AS, *s* will be pure action 1 and ns pure action 2. Using all these concepts, (26) becomes:

(28) $$\begin{array}{c} \left(1-\delta \right)\left\{\left(\alpha_{c}+\alpha_{m}\right)\phi_{11}-\alpha_{f}\phi_{12}\right\}+\delta \left(V_{1,n}-V_{1,o}\right)\left(\phi_{11}+\phi_{12}\right)\leq 0\\ \left(1-\delta \right)\left\{\left(-\alpha_{c}-\alpha_{m}\right)\phi_{21}+\alpha_{f}\phi_{22}\right\}+\delta \left(V_{1,n}-V_{1,o}\right)\left(\phi_{21}+\phi_{22}\right)\leq 0\\ \left(1-\delta \right)\left\{-\beta_{s}\phi_{11}+\beta_{c}\phi_{21}\right\}+\delta \left(V_{2,n}-V_{2,o}\right)\left(\phi_{11}+\phi_{21}\right)\leq 0\\ \left(1-\delta \right)\left\{\beta_{s}\phi_{12}-\beta_{c}\phi_{22}\right\}+\delta \left(V_{2,n}-V_{2,o}\right)\left(\phi_{12}+\phi_{22}\right)\leq 0, \end{array}$$

where we assumed that ϕ(nd)>0, ϕ(d)>0, ϕ(s)>0 and ϕ(ns)>0. The restrictions on the joint probability distribution ϕ (i.e., all components are non-negative and add up to 1) and the payoff that each player would obtain by following UNR strategy, obtained doing the mathematical expectation on ϕ of the payoffs in (8) are:

(29) $$\begin{array}{cc} \phi_{11} & +\phi_{12}+\phi_{21}+\phi_{22}=1\\ 0\leq & \phi_{ij}\leq 1,\;i\in \left\{1,2\right\},\;j\in \left\{1,2\right\}\\ V_{1,o} & =-\alpha_{m}\phi_{11}+\alpha_{c}\phi_{21}-\alpha_{f}\phi_{22}\\ V_{2,o} & =\beta_{s}\phi_{11}-\beta_{c}\phi_{21}. \end{array}$$

The expressions in (28) and (29) define the region of CE and the payoffs that players can obtain in the repeated game.

## 7. CSMA/CA Repeated Game with an Arbitrary Number of Players

The analytical derivations from the previous Sections may become intractable when there are many players. For these cases, we propose using CA (Communicate and Agree), which is a distributed algorithm to negotiate in repeated games using simple strategies described in Reference [30]. CA is based on the players communicating each other possible equilibrium points and accepting or rejecting them. It requires a stage equilibrium as input which CA tries to improve using repeated game theory tools, that is, the Folk Theorem and outputs a Pareto-efficient repeated game CE or SPE, as CA works with both equilibrium conditions. We implement CA using UNR as strategy, as in the two player case. In order to obtain the input stage NE, we use RM algorithm, presented in Section 5.3. We note that CA is specially suited for our problem because it is fully distributed and does not need that a central entity controls the negotiation, it explicitly uses the Folk Theorem as we use CA with conditions (16) and (18) for SPE and (28) for CE and it needs that each player knows only its own payoff function but not the ones of the rest of players.

CA algorithm conducts a negotiation prior to starting the play and this negotiation has two main phases: an action-space sampling and a pruning procedure. During the sampling phase, each player samples the action space *A* trying to find strategies which are equilibrium points for her. This means that, in case of SPE, the server samples trying to find points that satisfy (16) and each AS tries to satisfy (18); and in case of CE, each player tries to fulfill condition (28). Note that each player tries to find an equilibrium point for herself, as players need not knowing the payoff functions of the rest of agents.

When a player finds a candidate equilibrium point, that is, a vector of actions that is an equilibrium for her, she communicates this equilibrium to other players, who check whether this point is also a valid equilibrium for them or not. If the point is a valid equilibrium for all players, where again, we note that each player only checks whether it is an equilibrium for her, the equilibrium point is added to a list of candidate equilibrium, $A_{s}$; otherwise, the point is dropped. Note that the main idea of this procedure is that players try to find, in a distributed fashion, a set of valid equilibrium points for all players, $A_{s}$.

There are several sampling procedures proposed in Reference [30] and we use the one that provides best results in that work—an intelligent sampling schema based on Stochastic Optimistic Optimization (SOO) [31], which is a non-convex optimization algorithm. This method allows finding good candidate equilibrium points with few communications among players, at a higher computational cost. In order to bound this cost, the sampling phase is limited to a maximum number of communications per player, $N_{c}$, that is, a player can propose a maximum number of equilibrium points to the rest.

When the communication phase has finished, it may happen that $A_{s}$ is empty, which means that no valid equilibrium for all players has been found. In this case, the stage equilibrium provided as input is returned, because CA did not find a better equilibrium. However, if $A_{s}$ contains equilibrium points, a second phase starts, in which a pruning procedure is used in order to distributedly choosing a Pareto-efficient equilibrium, so that no player is allowed to dominate others when choosing the equilibrium point. We note that we use CA combined with UNR strategy, although other strategies could be used as well in CA [30]. The whole procedure is summarized in Algorithm 1.

Finally, we recall again that RM algorithm described in Section 5.3 does not learn a repeated game equilibrium using the tools provided by the Folk theorems. RM can be used for learning equilibria in repeated games, since static NE and CE are equilibria of the repeated games. But stage equilibria payoffs need not be the best payoffs that players might achieve: the main reason to use the Folk theorems tools is that they allow providing all players with a payoff strictly higher than the ones they obtain by following a static strategy. While RM does not make use of the Folk Theorem, CA does make use of the Folk Theorem tools, as the equilibrium condition that must be satisfied for all players is based on Proposition 1.

**Algorithm 1** CA algorithm for each player *i***Input:**$\delta_{i}$, $u_{i}$, $a_{i,n}$, $u_{i,n}$, $N_{p}$, $N_{c}$ 1: $A_{s}\leftarrow sample-actions\left(\delta_{i},u_{i},a_{i,p},u_{i,p},N_{p},N_{c}\right)$ 2: **if**
$A_{s}=\emptyset$
**then** 3:  $A_{s}=a_{i,p}$ 4: **else** 5:  **while**$|A_{s}|>1$
**do** 6:   $A_{s}\leftarrow pareto-prune\left(A_{s},u_{i}\right)$
**Output:**
$A_{s}$


## 8. Simulations for the CSMA/CA Game

In order to validate the theoretical developments in the previous Sections and observe how the different solutions proposed perform in practice, we perform some simulations on a wireless network. We fix the number of stations to n=5, we use BA mechanism and $T_{p}=8184$ bits in order to estimate the network throughput using Bianchi’s model. The parameters of NSs, denoted by subscript 1, are $W_{1}=32$, $CW_{max,1}=1024$ and hence, $m_{1}=5$. The ASs, denoted with subscript 2, use the uniform random mechanism, with a window length $W_{2}=8$. The rest of IEEE 802.11 parameters are in Table 2, which are used in References [15] and [8]. With these values, we solve (1) to (5) to obtain the throughput values for $n_{2}\in \left\{1,2,3,4\right\}$. The parameters of the payoff matrix from Table 1 are $k_{s}=k_{c}=1$, $k_{d}=0.1$. With these values and the results of Bianchi expressions, we can obtain the payoff functions for a given number of ASs. For instance, when $n_{2}=1$, we obtain $S^{ns}=0.1617$, $S_{n}^{s}=0.0700$, $S_{c}^{s}=0.5225$, which gives rise to the payoff matrix in Table 3, that is used in our simulations.

### 8.1. Simulation 1: Dependency with δ

**Figure 2:**
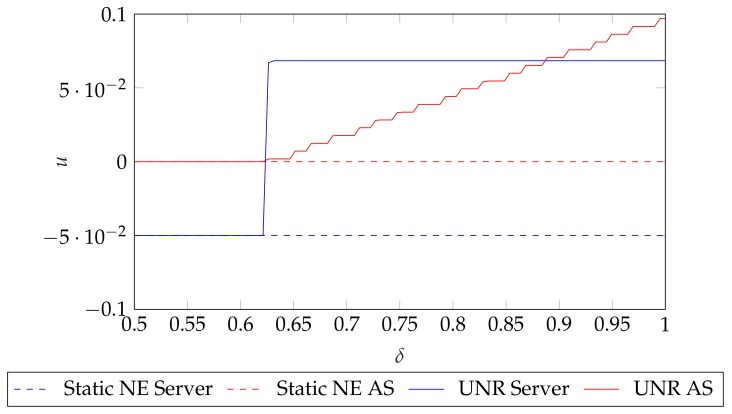
Maximum payoff *u* that the server and AS could obtain in the two-player case as a function of δ using Unforgiving Nash Reversion (UNR). Note that the conditions from (20) are only fulfilled when δ surpasses a certain threshold. Note that the Folk Theorem conditions are satisfied only as δ→1 and the concrete threshold on δ depends on the concrete game payoffs.

First, we illustrate the influence of the value of δ on the best payoffs that each player could obtain in the two player case, whose payoff matrix is collected in Table 3. First, we obtain the static Nash values using (9) and (11). Then, by making use of UNR strategy, we sample *y* and *z* uniformly using 10,000 samples in the unit square $\left(y_{o},z_{o}\right)\in \left[0,1\right]\times \left[0,1\right]$ and check the conditions from (16) and (18) for each $\left(y_{o},z_{o}\right)$ pair, in order to check whether they are valid equilibria. We repeat the whole procedure for 100 δ values equispaced in the range δ∈[0,1] and the results are shown in Figure 2. Note that we show the maximum payoff that each player could obtain such that it satisfies (16) for the server and (18) for the AS. As the Folk Theorem advances, there is a minimum δ value that allows players obtaining better payoffs than the static NE. Hence, by having discount factors close to 1, both players are able to achieve better payoffs.

### 8.2. Simulation 2: Repeated CSMA/CA game solutions

In the previous Simulation, we have shown the maximum payoffs that players could obtain as a function of δ. Now, in order to compare the static and the repeated payoffs of the CSMA/CA game, we compare the solutions that RM provides with the solutions given by CA. We fix the discount factor value to δ=0.99, which as shown by Figure 2, allows both players to improve their payoffs. Also, note that 1−δ can be understood as the probability that each player assigns to the interaction finishing in the next stage, hence, we choose a δ value which assigns low probability to stopping the interaction, which suits our setup. For CA, we set $N_{c}=100$ communications per player.

As sampling procedure, we have used SOO [31]. Since SOO samples in a hypercube, this is appropriate for the SPE case: we have two actions per player, hence, the mixed actions vector for $N_{p}$ players will lie in the hypercube of dimension $N_{p}$, whose components lie in the range [0,1], that is, the mixed actions vector *a* is so that $a\in {\left[0,1\right]}^{N_{p}}$. However, the CE solution is a distribution ϕ that has, in our case, $2^{N_{p}}$ components. It must satisfy that $\phi_{k}\geq 0$ and $\sum_{k}\phi_{k}=1$ and hence, it is a simplex, not a hypercube. This means that, as $N_{p}$ grows, if we sample a hypercube, we will lose a lot of points because they do not belong to the valid region of the distribution ϕ. In order to solve this problem, we use a mapping from a hypercube to the simplex region containing ϕ. For a vector *x* that belongs to the hypercube of dimension $N_{p}-1$, we compute $s=\sum_{k}x_{k}$ and $m=\max(x_{k})$ and obtain the point $x^{\prime }$ as follows:

(30) $$x^{\prime }=x\frac{m}{s},$$

where $x^{\prime }$ satisfies that $x_{k}^{\prime }\geq 0$ for its $N_{p}-1$ components and $\sum_{k}x_{k}^{\prime }\leq 1$. Hence, we can define a candidate equilibrium distribution $\phi_{c}$ as:

(31) $$\phi_{c}=\left(x_{1}^{\prime },x_{2}^{\prime },\ldots ,x_{N_{p}-1}^{\prime },1-\sum_{k}x_{k}^{\prime }\right),$$

where we recall that $x^{\prime }$ was obtained from the hypercube of dimension $N_{p}-1$. By doing this we ensure that $\phi_{c}$ satisfies the conditions to be a valid distribution.

Sampling using SOO has a λ∈[0,1] parameter [30], which models the selfishness of a player. We simulate using λ=1, that is, the player ignores the rest of the players and λ=0.5, that is, the player takes into account the rest of players. Also, for the SPE, we must define a grid of actions to test for deviations; in our case, we provide a uniformly distributed grid in the range [0,1] with 30 samples.

**Figure 3:**
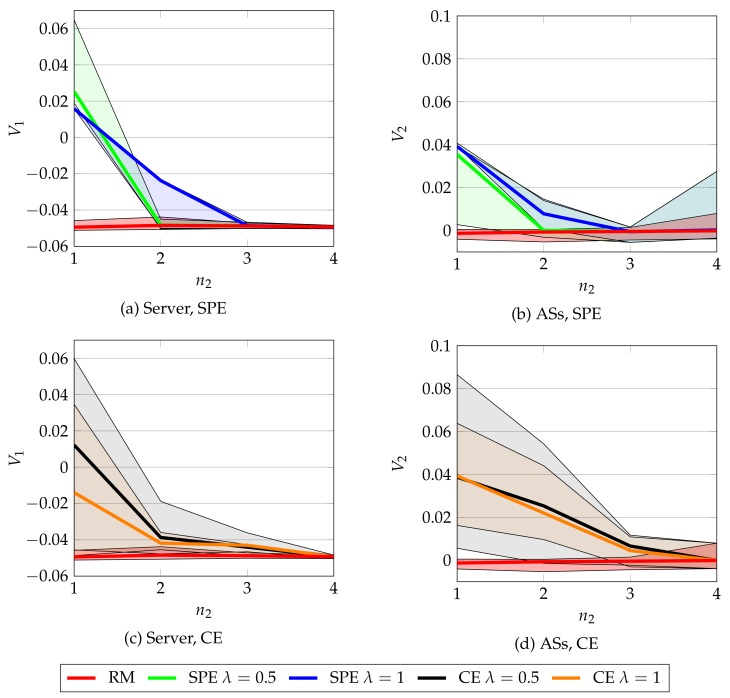
Payoff *V* obtained for the server and AS, using CA. The error bars show the maximum and minimum values achieved. For ASs, we plot the mean values, computed among the $n_{2}$ ASs in the setup. We can observe that CA never performs worse than RM and when there is a low number of ASs it provides a significant payoff gain to both server and ASs.

We test CA for both CE and SPE concepts, using λ={0.5,1} and for n=5 stations in the network. We consider that $n_{2}=\left\{1,2,3,4\right\}$. For each of these cases, we first obtain a static equilibrium using RM algorithm with T=2000 iterations and the results of RM are given as input to CA algorithm. After CA algorithm has been run, we obtain a possibly higher payoff. We repeat 50 times the whole procedure for each $n_{2}$ value and the results are in Figure 3. Observe that (1) as expected by design, CA never provides a lower payoff than RM, (2) the payoff increases are bigger and with higher variability when $n_{2}$ is lower, that is, when there are fewer ASs, (3) CE and SPE provide similar results, with an advantage for CE in the case of the ASs and (4) the payoff gains are smaller for the ASs than for the server.

**Figure 4:**
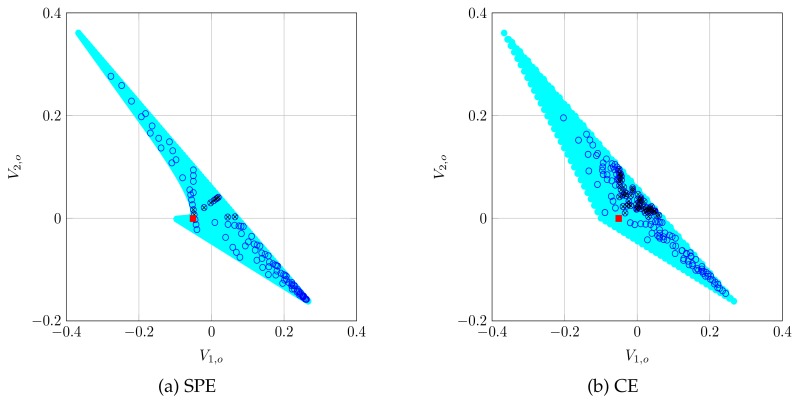
Payoff region when $n_{2}=1$, using SPE and CE. The light region are all possible payoffs, the red square is the static NE that RM provides, the blue circles are the points that CA samples and the circles with a black cross are those that are valid equilibria for the repeated game, that is, there is a greater payoff for both players than their stage NE payoff. Observe that the SPE region is contained in the CE region.

Finally, a representation of the payoff regions can be observed in Figure 4 for both SPE and CE, for the case in which $n_{2}=1$, using the expressions derived in Section 6. Observe that the region of valid payoffs (i.e., those which yield a greater payoff than the static NE) is not too large. This explains why, in Figure 3, the increments in payoffs that CA returned were small: they cannot be too large due to the characteristics of the payoff region. Note that this Figure also explains why the results in Figure 3 are far from the maximum values that players could obtain, as shown by Figure 2: a high payoff for the server means a lower payoff for the AS and the other way around; hence, they must compromise between their maximum possible payoffs and improving their static NE payoffs. As shown by Figure 3, they succeed in this task.

### 8.3. Simulation 3: Computational Resources of RM and CA

**Figure 5:**
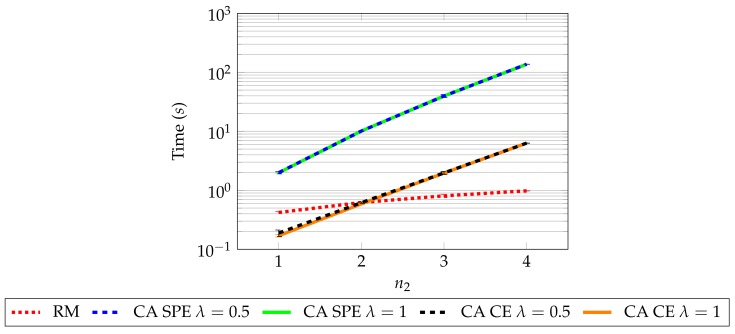
Time consumed computing an equilibrium using different values of $n_{2}$ for all the algorithms tested. It is possible to observe that RM provides the best scaling, whereas all CA algorithms scale worse. Also, observe that the CE version of CA requires significantly less time than the SPE to be computed: it takes around one order of magnitude less time. This is to be expected, as CE are more efficient to compute than NE [23,24].

Another aspect to take into account is the computational resources required by each algorithm. We obtain the mean execution time for the cases in the previous simulation, which can be observed in Figure 5. All the scripts were programmed in MatLAB^®^, without parallelization and run on a computer having an Intel i7-950 processor, clocked at 3.06 GHz and accompanied by 20 GB of RAM. For these purposes, we do not measure the time that would take to the stations to communicate among them: this increment on time would be dependent on the concrete communication procedure used. Rather, we center on the computational time required to run RM and CA.

The results in Figure 5 show that RM presents the best scaling as the number of players increases. Regarding CA, the value of λ does not make a significant difference but the equilibrium type does: CE is around one order of magnitude below SPE and thus, CE is significantly faster to compute, as expected [23,24].

Observe also that all CA variants present an increase of computational requirements exponential with the number of players. This means that CA may not be the best option with a large number of players. As shown in Reference [30], the communication phase among stations can be done efficiently in polynomial time. However, there are two main problems that may make CA inefficient with a large number of agents. The first one is the computational load of the sampling method used: as we note, we use an intelligent sampling procedure which, however, is computationally expensive. The second is the fact that the action space dimensionality grows with the number of agents. Thus, further research is needed in order to figure out whether CA scalability can be improved, specially when dealing with large scale networks.

Finally, recall that for each case in which CA is run, we must feed it with a static NE. We proposed using RM for this task; hence, the total CA computation time is formed by adding to each CA value in Figure 5 its corresponding RM value.

### 8.4. Discussion

The results of the previous simulations have an impact on practical implementations of the defense mechanism proposed. The first question is whether to implement a static or repeated game solution. We have shown, in Figure 3, that the repeated solution might provide higher payoffs to all players. This increment, as shown in Figure 4, is significant in terms of the payoff region. But this payoff gain comes at the cost of more computational resources: Figure 5 shows that RM scales better in terms of computational resources than CA. We also must take into account that CA requires a stage NE as input, so it can be thought of as an additional cost after having a stage NE. In short, there is a trade-off between computational time and payoff gain. If we are more interested in have a low computational time, as may be the case in a sensor network with low computational resources or large constraints in battery life, then static equilibrium might be the more sensible option.

If we decide to use a repeated solution based in CA algorithm, then two more questions arise. The first is related to the concrete parameters of the algorithm to use: λ, $N_{c}$ and the sampling procedure. These parameters have an effect on the equilibrium that CA returns as shown in Reference [30]; and hence, we have to find a set of parameters that performs adequately in our concrete setup, as a function of the computational resources, the network topology and the payoff gain desired.

We observe that CE is preferable to SPE for different reasons. First, Figure 3 shows that CE performs similarly in terms of payoff gain. Second, Figure 4 shows that SPE region is contained into the CE region, so any Nash equilibrium will have a corresponding correlated equilibrium but the reverse is not true. Third, Figure 5 shows that CE is significantly faster to compute. However, CE is based on a correlating device, which obtains realizations of the equilibrium distribution ϕ and sends the action to play to each player. For instance, in the context of IEEE 802.11, this task could be performed by the HCF (Hybrid Coordination Function), a centralized network coordinator whose task in this case would be obtaining realizations of the distribution ϕ and sending them to each player. Note that CE reminds of a centralized scheduler such that no station gains by deviating from its recommendations.

Finally, we have derived equilibrium conditions which are valid only in a perfect monitoring environment. This means that players are able to detect deviations instantaneously. In the case of CE, this is straightforward: the correlating device, in each stage game, sends each player the pure action that she should play: if any player deviates, the correlating device would know at the end of that stage. The case of SPE is much harder: players play mixed strategies, which mean that the other players can detect a deviation instantaneously only if they have access to the correlating device of the rest of the players. This might not be practical in terms of implementation and it is another reason to see CE as superior to SPE in practical terms.

## 9. Conclusions

In this article, we study a CSMA/CA wireless network under a backoff attack: some stations deviate from the defined contention mechanism and this causes the network throughput not to be fairly distributed. This impact is studied using Bianchi’s model and posed as a game. We first solve this game using static solution concepts and then we use repeated game tools in order to take into account the fact that there is more than one transmission in the network. We first provide an analytical solution to the repeated game in the two player case, using both CE and SPE equilibrium concepts and then we also propose an algorithm that can be used to distributedly obtain repeated game equilibria. By using simulations, we are able to check that using repeated game tools allows the players to have better payoffs and we also study the computational cost required by each of the solutions we compare.

There are several ways in which this work could be continued. First, it would be possible to obtain the payoff regions for different repeated game strategies: in this work, we have only used UNR but as we mention, there are many others that could be used and each of them potentially may give different payoff regions. Second, we have considered that the server is able to detect perfectly a deviation without error, however, such ideal detectors do not exist in the real world. It would be interesting including the effect of the error in the detection in the game analysis, however this may significantly modify the analytical tractability of the problem. Third, there is a significant margin to improve the scalability for the case in which there are many agents and, as we have indicated, it would be important comparing variants of CA in terms of scalability with the number of agents. And lastly, we have considered that there is perfect monitoring, in that each player can observe the actions of the rest of the players at the end of each stage. This assumption may not be true in all situations and hence, a partial monitoring schema could be another way to continue the present work.

## Figures and Tables

**Table 1:** Payoffs values for the CSMA/CA game when $n_{2}=1$. The payoff vectors are of the form $u=(u_{1},u_{2})$, where $u_{1}$ is the payoff of the server and $u_{2}$ is the payoff of the AS. All *k* constants are positive. $-k_{d}$ is the cost of detecting an attack for the server. $k_{s}$ is the cost that the server incurs for not detecting an attack. $k_{1}$ denotes the gains of the AS if it increases its bandwidth share. $S^{ns}$ is the throughput that each normal station (and the AS) obtain if the AS plays ns. $S_{n1}^{s}$ / $S_{1}^{s}$ is the throughput that each normal station / the AS obtain if the AS plays *s* and it is not detected. Note that $S_{n1}^{s}<S_{1}^{s}$, which means that the AS has an incentive to behave selfishly.

	*s*	ns
nd	$\left(k_{s}n_{1}\left(S_{n_{1}}^{s}-S^{ns}\right),k_{1}\left(S_{1}^{s}-S^{ns}\right)\right)$	$\left(0,0\right)$
*d*	$\left(k_{s}n_{1}\left(S^{ns}-S_{n_{1}}^{s}\right)-k_{d},-k_{1}S^{ns}\right)$	$\left(-k_{d},0\right)$

**Table 2:** IEEE 802.11 simulation values.

MAC Header	272 bits	$T_{\delta }$	1 μs
PHY header	128 bits	$T_{s}$	50 μs
ACK	112 bits + PHY header	SIFS	28 μs
RTS	160 bits + PHY header	DIFS	128 μs
CTS	272 bits + PHY header	Bit rate	1 Mbps

**Table 3:** Payoffs values for the game when $n_{1}=4$ and $n_{2}=1$, that is, there are n=5 stations and only one of them may attack. The first entry of the payoff vector is the server payoff, the second is the AS payoff.

	*s*	ns
nd	$\left(-0.3668,0.3608)\right)$	$\left(0,0\right)$
*d*	$\left(0.2668,-0.1617\right)$	$\left(-0.1,0\right)$

## Abbreviations

Main abbreviations and symbols used in this manuscript:

*A*/$A_{i}$	Set of actions available to all players/to player *i*
AS	Attacking Station
CA	Communicate & Agree
CE	Correlated Equilibrium
CSMA/CA	Carrier-Sense Medium Access with Collision Avoidance
CW	Contention Window
DCF	Distributed Coordination Function
δ	Discount factor
ϕ	Correlated equilibrium distribution
*m*	Maximum backoff stage
MAC	Medium Access Control
*n*	Number of stations in the network
$n_{1}$	Number of normal stations in the network
$n_{2}$	Number of attacking stations in the network
$N_{p}$	Number of players
NE	Nash Equilibrium
NS	Normal Station
$p_{i}$	Probability that station *i* observes a collision
$R_{i}$	Payoff matrix for player *i*
RM	Regret Matching
$S_{i}$	Throughput for station *i*
SPE	Subgame Perfect Equilibrium
$\sigma_{i}$	Strategy for player *i*
*t*	Time index
$\tau_{i}$	Probability that station *i* transmits
*u*	Game payoff function
UNR	Unforgiving Nash Reversion
$V_{i}$	Average discounted payoff for player *i*
*W*	Minimum size of the contention window
*y*	Mixed action of player 1
*z*	Mixed action of player 2
